# Innovative vanillin yielding from lignin: process modelling and assessment

**DOI:** 10.12688/openreseurope.16734.2

**Published:** 2024-12-24

**Authors:** Álvaro Cabeza Sánchez, Olaf Trygve Berglihn, Eloy Ottaviano, Theresa Rücker, Torbjørn Pettersen, Bernd Wittgens, Abraham Aliko, Lucía Gálvez, María López

**Affiliations:** 1 BIOTECH, IDENER RESEARCH AND DEVELOPMENT AIE, LA RINCONADA, SEVILLE, 41300, Spain; 2 Process Technology, SINTEF Industry, Trondheim, Trøndelag, NO-7465, Norway; 3 ENSO INNOVATION, CULLEREDO, A CORUÑA, 15180, Spain

**Keywords:** Lignin; Vanillin; Electrochemical; Depolymerisation; TEA; LCA; Model

## Abstract

In this work, we present the modelling of a novel process to promote vanillin production from lignin using PODIC ^®^. The model includes the PODIC® production, lignin depolymerisation, and final product separation. Furthermore, a complete assessment of the proposed process in terms of economic and environmental performance was carried out. Regarding the economic evaluation, a comprehensive study was done leading to high investment (13.76 €/kg lignin) and operational (5.16 €/kg lignin) costs. The main reasons for the elevated costs were identified and alternative process configurations were evaluated. Despite this, the main result was that the proposed process is challenging to commercialize. An environmental analysis of the environmental impacts generated by the overall production process of vanillin and phenolic compounds was carried out considering energy consumption, human health, greenhouse gas emissions, water consumption and human toxicity. Electricity consumption of the PODIC ^®^ reactor was identified as the hotspot of the system. The CO _2_ emissions were also compared with the *Borregaard* process and found to be similar.

## Introduction

Vanillin is a versatile compound with a plethora of feasible applications in different industries like (1) the food industry (as flavouring), (2) the cosmetic industry (as fragrance), (3) the chemical industry (as an antifoaming agent), and (4) pharmaceutical industry (as odour-masking agent) ^1^. The foreseen global production in 2024 is expected to be close to 60,000 t (a growth of 8% from 2019) ^2^.

There are several routes to produce synthetic vanillin, but the most prominent option is the petroleum-based route, specifically from guaiacol (80% of the world's supply) ^3– 5^ while natural vanillin accounts for just 1% of the total market ^6^. The rest is mainly obtained by a bio-based pathway through oxidative depolymerisation of lignin/lignosulfonates ^7^. *Borregaard* (Norway) is the main active vanillin producer from lignosulfonates, operating for the last 20 years using this approach ^3^. *Borregaard* obtains vanillin inside a multiproduct biorefinery with spruce wood as feedstock. Focusing on the vanillin process, it is fed by the liquor generated as waste during the sulphite process of wood pulp. Lignin depolymerisation into vanillin consists of oxidant treatments at alkaline conditions, high temperatures and high pressures. The first stage generates concentrated lignosulfonates using ultrafiltration, precipitation, and evaporation. After the reaction phase, the product mixture is separated by liquid-liquid extraction. During the depolymerisation of lignin, a heterogeneous mixture of compounds is produced, containing aldehydes, acids and phenolics, among other byproducts. High-purity vanillin can be obtained when this mixture is treated by a sequence of separation and purification stages. Furthermore, to promote vanillin production, copper-based catalysts are used ^8^ although it also means high complexity in the reaction stage since it implies a three-phase reactor.

The previous process focused on lignosulfonates but Kraft-lignin valorisation can be performed by a similar approach, which is highly interesting since its production is larger and more cost-efficient than the one for lignosulfonates ^3, 9^. Valorisation by acidic oxidation seems to be also feasible, but the development of this approach is still ongoing with a low TRL ^10^. Indeed, different works have been published in the literature dealing with lignin depolymerisation by alternative technologies like biological, microwave-assisted, pressurized fluids, ionic liquids, electrochemical, or deep eutectic solvent depolymerisation ^11– 14^. Among them, the electrochemical pathway is especially relevant since it only requires electricity to drive the depolymerisation reaction. Thus, it avoids the need for severe operational conditions and/or harmful chemicals, also enhancing the direct integration options with renewable energy, which potentially increases the sustainability of the lignin depolymerization. For this reason, the electrochemical pathway was deeply studied following two different approaches, via an ex-cell ^14– 16^ oxidiser and direct ^17– 21^ electro-depolymerisation. An ex-cell produced oxidizer has the advantage of being used as a platform chemical of which the properties can be customized individually. For lignin depolymerization, this oxidizer can be mixed with lignin for pre-oxidation, which will be followed by a thermal treatment. Here, the lignin can be dissolved in a sodium hydroxide solution or used directly as a powder. The latter option has a decisive economic and environmental advantage, as it does not require the use of sodium hydroxide.

Although high selectivity is one of the main aims, a complex mixture is the usual product from any lignin depolymerisation reaction. Thus, vanillin production from biomass or lignin requires several purification steps due to the formation of a complex mixture of organics during depolymerization, which compromises profitability. Indeed, several studies have been performed with the objective to optimise the downstream separation, involving the use of membrane-based units and ion exchange recovery systems ^22, 23^. Nevertheless, it is not obvious if these modifications would be enough to ensure a positive cash flow for the operation. Therefore, improvements in the conversion steps are required to increase the vanillin yield. Furthermore, a profitable conversion of biomass requires the production of multiple products based on all three main compounds of woody biomass cellulose, hemicellulose and lignin, like in *Borregaard’s* case. Finally, profitability will be more complex in the case of Kraft-lignin as the vanillin yield is lower than the one found for lignosulfonates ^24^.

For these reasons, a completely new ex-cell approach was proposed in the H2020 project LIBERATE (grant agreement No 820735). The aim is to develop a solution that uses a liquid-phase oxidant, avoiding three-phase depolymerisation reactors. This oxidant will be electrochemically produced and, therefore, reducing the need for harmful chemicals while focusing the energy consumption on green electricity and increasing its sustainability. The selected oxidiser was sodium peroxodicarbonate, Na _2_CO _6_, PODIC ^®^ since it also promotes vanillin production ^14– 26^. Moreover, focusing on Kraft-lignin, the proposed process considered two different approaches. The first one followed a conventional operational way, where lignin is firstly dissolved in NaOH before being mixed with the PODIC ^®^ solution, which is produced from sodium carbonate and electricity in an electrochemical reactor. Further, the mixture is fed into a thermal reactor to perform the depolymerisation. The second approach explores a more innovative option where the lignin powder is directly dissolved into the PODIC ^®^ solution. This way, the use of external NaOH is prevented, being substituted by a cheaper chemical (sodium carbonate) and electricity that can be obtained from renewable energy sources. This alternative was developed as a solution to the negative economic performance of the first approach since it highly reduced the dilution and the need for external NaOH. Nevertheless, although the second LIBERATE solution was promising, it required a deep assessment in terms of economic and environmental performance, which is the main aim of the research gathered in this work. So, a comprehensive calibrated process model of the solution is presented, followed by a complete Techno-Economic Analysis (TEA) and Life Cycle Assessment (LCA). Further, the potential benefits in environmental impact terms can be considered by avoiding the current state of the art for lignin processing, which is combustion as biomass ^27^. This would reduce CO _2_ emissions, extracting value-added products from the lignin.

## Proposed solution

### Experimental background

The work displayed in this paper focuses on the modelling and the evaluation of the solution developed inside the LIBERATE project. Thus, no detail about the experiments performed will be provided. However, the experimental base can be found in the work of *Zirbes et al. ^14^*, where it is demonstrated that vanillin yields up to 6.2% are feasible from lignin thermal oxidation (at 180 °C) using Na _2_CO _6_ as an oxidiser.

On the other hand, the LIBERATE experimental activities did not only focus on the lab scale but also a pilot was developed ^28^, obtaining similar yields in both scales (up to 8%). Thus, it was demonstrated that the technology could be used at different scales without significant losses in the yields.

### Developed process

**Figure 1.**
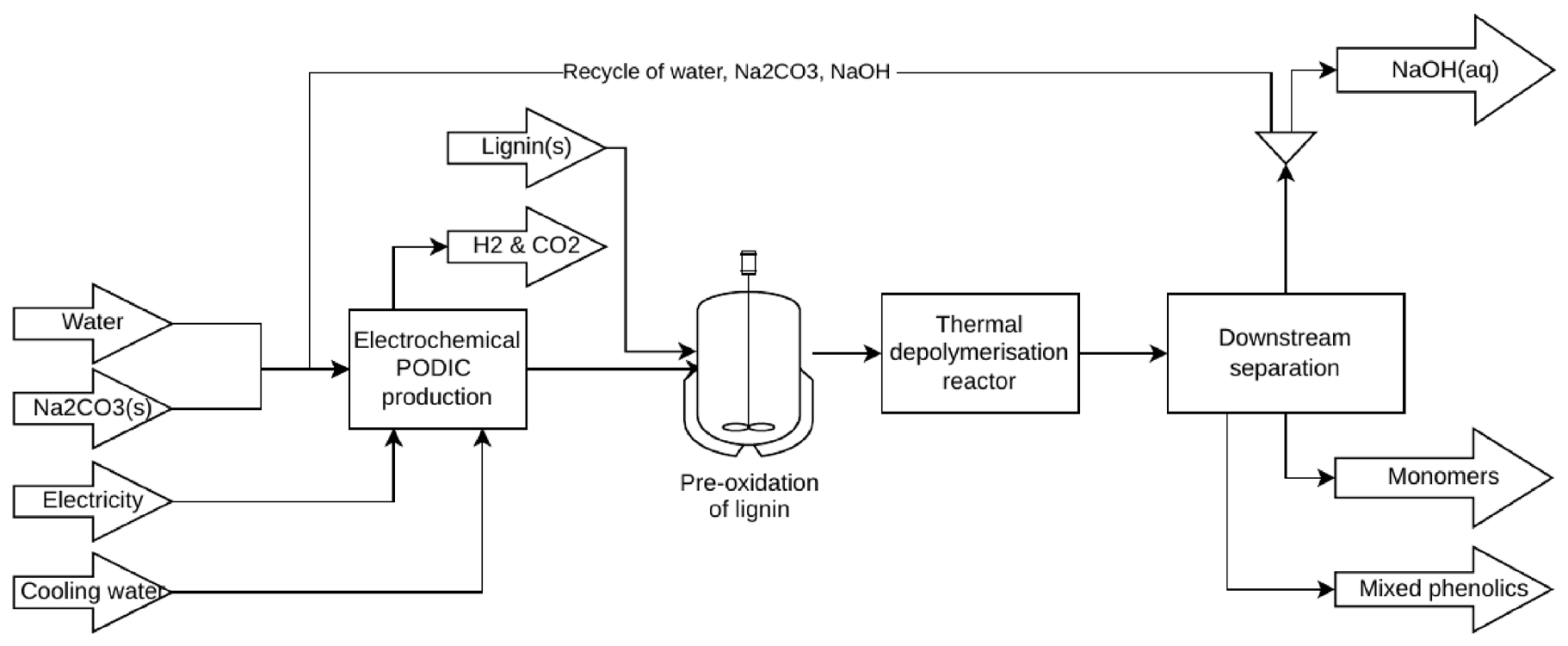
Process scheme.

The process consists of three main sections: 1) a PODIC ^®^ reactor converting sodium carbonate to peroxodicarbonate and sodium hydroxide by applying an electric current over boron-doped electrodes, 2) a reactor section where PODIC ^®^ solution is mixed with solid lignin to oxidize and thermally depolymerize lignin and produce the phenolic aldehydes, phenolic acids and guaiacol, 3) a product separation sequence where vanillin and light phenols are separated by an adsorber system and subsequent membrane section where the eluate from the adsorber section containing mixed phenolics and partially converted lignin is dewatered. NaOH and sodium carbonate follows the water in the membrane section and is recirculated to the PODIC ^®^ reactor. Sodium carbonate is required to produce the PODIC ^®^, and NaOH and CO _2_ are side products of this reaction. Thus, by sparging the produced CO _2_ during the decomposition in a downstream gas-liquid reactor also fed with the side the NaOH solution, the sodium carbonate can be regenerated (CO _2_(g) + 2 NaOH(aq) → Na _2_CO _3_(aq) + H _2_O(l)). For this reason, an alternative process with the addition of sodium carbonate regeneration was also established, which also includes the use of additional CO _2 _to enhance the regeneration. A scheme of the process is given in Figure 1 (see Energy and mass balances from the process model in Annex 1 for a more detailed scheme). 

The process was modelled in the COCO-simulator ( https://www.cocosimulator.org/) and using the CAPE-OPEN *Python* unit operation add-on, both by *Amsterchem*. The *Python* unit operation add-on was used with custom program code to describe the membrane section. The flowsheet based on Figure 1 can be found in the provided COCO-simulator file. Note that this flowsheet includes process integration actions, like sodium carbonate regeneration and recirculation. Indeed, this process flowsheet was the result of different interactions focusing on minimising the main sustainability issues early detected: dilution and PODIC ^®^ production cost. The complete set of simulator input files and auxiliary codes are given at ( https://doi.org/10.5281/zenodo.8424715) ^1^.

### PODIC ^®^ production pathway

As previously mentioned, the process concept developed inside the LIBERATE project to maximize vanillin production is based on the use of PODIC ^®^ (Na _2_CO _6_). Thus, the first step in the proposed solution is the production of the liquid oxidiser (PODIC ^®^) to use in the thermal reactor. To do so, a preliminary electrochemical reactor (using a boron-doped anode and a stainless-steel cathode) is needed to perform its production from Na _2_CO _3_. More details can be found in the piloting work ^28^.

Thus, to include it in the assessment, its production from sodium carbonate had to be modelled. The net reaction from sodium carbonate to sodium peroxydicarbonate (PODIC ^®^) is assumed to be:

2 Na _2_CO _3_ + 2 H _2_O → Na _2_C _2_O _6_ + 2 NaOH + H _2_ (g)

The PODIC ^®^ will also decompose during the operation, and a plausible mechanism for the decomposition is given by:

2 Na _2_C _2_O _6_ + 4 H _2_O → 2 H _2_O _2_ + 4 NaHCO _3_

With the peroxide eventually decomposing by:

2 H _2_O _2_ → 2H _2_O + O _2_(g)

So, the net overall reaction becomes:

2 Na _2_C _2_O _6_ + 2 H _2_O → 4 NaHCO _3_ + O _2_ (g)

The formation of bicarbonate will affect the carbonate equilibrium in the electrolyte and may lead to the release of carbon dioxide if the electrolyte is saturated with CO _2_ according to the following reactions:

NaHCO _3_ + H _2_O ⇄ H _2_CO _3_ + NaOH

H _2_CO _3_ → H _2_O + CO _2_(g)

Thus, based on the assumption of CO _2_ saturation in the electrolyte, the overall reaction for PODIC ^®^ decomposition is formulated as:

2 Na _2_C _2_O _6_ + 2 H _2_O → 4 NaOH + 4 CO _2_ + O _2_(g)

### Lignin depolymerisation mechanism

In order to represent a possible varying lignin composition, the lignin was parameterized as the lignin monomers in their alcohol representation: p-coumary alcohol, coniferyl alcohol, and sinapyl alcohol. Of primary interest are the phenolic aldehydes, phenols and quinones that can be derived from these:

- Hydroxy-benzaldehyde, vanillin and syringaldehyde, acetovanillone, and acetosyringone
- Various phenols
- Ring-oxidated compounds of the above.

Thus, the reaction mechanism was based on simplified reactions (monomer to product + residue), defining the required stoichiometries to close the mass balance. Note, naturally this is a simplified description of the complex substrate, the reactions, and its product mixture, though it is a sufficiently accurate description with respect to the purpose of modelling the full process, utility systems, and separation system. The overall aim is to develop a simplified but closed mass balance for the main classes of products, the energy costs, and the sizing and definition of equipment. **The different reactions can be found in the developed flowsheet (available at https://doi.org/10.5281/zenodo.8424715), constituting the main output from this section.**

### Kinetics and thermodynamics

For PODIC ^®^ production, the forward reaction rate to sodium peroxodicarbonate can be calculated by knowing the number of electrons transferred and, the current and an efficiency factor:


$$r_{1}=I\cdot \eta /(v\cdot F)$$

Where I is the current, η is the current efficiency, ν is the charge count and F is the Faraday constant. The reverse reaction of sodium peroxodicarbonate decomposition can be represented by a standard Arrhenius expression and as a first-order reaction


$$r_{2}=V\cdot A\cdot \text{e}(\frac{-E_{a}}{RT})\cdot C$$

where V is the reaction volume and C is the concentration of PODIC ^®^, and the remaining expression is the classical Arrhenius equation.

The degradation reactions for lignin are modelled by specifying the degree of conversion. Since the temperature effect of the lignin degradation is negligible, the focus when defining the thermo-dynamic properties of the chosen components and the mixture of these is to get a proper description of the heat capacity and the vapor-liquid and liquid-liquid equilibrium for the main separation operations. The process includes an adsorption system using acetic acid and alcohols to strip the adsorbent and the thermodynamic description was chosen to be the Non-random Two-Liquid (NRTL)-model, using the Hayden O’Connel model for better describing the association effects in the gas phase caused by acids. The model parameters have been used as available in the *ChemSep* package database. Where the components were not already defined, parameters as given by NIST database have been used. Where no data was available in NIST the compounds have been given the correct gross molecular formula and otherwise been set to the same values as for vanillin.

## Process assessment

### Techno-Economic Assessment (TEA)

The process TEA was based on the process model described in the section Developed process, also assessing the two different working modes (with CO _2_ supply for NaOH conversion and without the feeding of extra CO _2_). The evaluated case is the same as the one used in the process model: a plant that treats 1,268 kg/h of lignin to produce vanillin, phenols, and partially depolymerized lignin.

Concerning the economic model, the bare module costing method ^29^ is used. This method estimates both capital (CAPEX) and operational (OPEX) costs based on average values for the chemical industry. To evaluate the purchase price of equipment components, the methodology considers both direct and indirect costs, as well as contingencies and fees. Additionally, when there is limited information, the method also includes correlations based on industrial equipment to compute the base purchase cost in base conditions (common materials and near-ambient pressures) and its correction by correlation factors to incorporate the equipment material and operational conditions (mainly pressure and chemical resistance). These correlations are based on the main characteristics feature of the evaluated device. The assumptions to estimate these features are listed in ANNEX 1, where the equations to compute the CAPEX and the OPEX are also included. Furthermore, scale-up robustness was demonstrated (see section Experimental background) and numbering up and engineering constraints (like maximum device size in an industrial environment) were imposed for the electrochemical reactor to have a realistic evaluation. The investment cost for each involved device calculation can be also seen in ANNEX 1. To compute the OPEX contributions, energy and mass balances were needed. These balances were obtained from the process model and the information retrieved can be found in ANNEX 1.

Finally, once the CAPEX and OPEX are known, the process's economic performance can be evaluated by several economic indicators, mainly the Net Present Value (NPV), the Internal Return Rate (IRR), and the Return on Investment (ROI). A detailed description of them is done in ANNEX 1.

***TEA tool***

The TEA was performed by a tailored tool developed in *Python* that can be found at https://doi.org/10.5281/zenodo.8424715. This tool and the computed economic indicators are the main results of this section.

### Life cycle analysis (LCA)

This study aimed to evaluate the environmental impact of the production process for vanillin and phenolic compounds, being these evaluations the main outcomes from this part. This calculation was carried out using *SimaPro 9.3.0.3* software, the *ReCiPe 2016* method (midpoint with hierarchical perspective), limiting the impact categories to be analyzed to 5 (energy consumption, human health, greenhouse gas emissions, water consumption and human toxicity) and secondary data obtained and adapted from Ecoinvent 3.8 database. The inventory is available at https://doi.org/10.5281/zenodo.8424715.

The study has a cradle-to-gate scope, the functional unit was defined as 1 kg of the desired product and the geographic and technological scope is the European one.

Life cycle analysis starts from the environmental impact associated with dry Kraft-lignin powder production ^30, 31^. For this environmental analysis, 3 subsystems were identified:

- Lignin Feed, using lignin derived from the Kraft process.
- PODIC ^®^ process considers the input of sodium carbonate and water, as well as the energy consumption of the reactor and the pumps (PODIC ^®^ and feed).
- Thermal Depolymerization + Separation: this subsystem considers the rest of the process.

Consumptions involved (varying between the case with CO _2_, WCO _2_, and without CO _2_, NoCO _2_), taking as an avoided product the production of low-concentration acetic acid. Also, for this subsystem a mass allocation was applied between the two products obtained: vanillin and phenolic compounds. More detailed information is provided in ANNEX 2.

### Process CAPEX and OPEX

***Analysis of the process with CO _2_ supply***

**Capital cost (CAPEX)**

The investment cost for each unit is listed in ANNEX 1, while the total CAPEX (once the corresponding multipliers to include piping, instrumentation, and other additional investment costs were included) was 145 M€, which is relatively high in comparison to the literature cases (see Table 3), with a distribution between the reaction and separation sections is close to 50%. A high CAPEX was not unexpected since the involved stream has a high dilution rate (water content >70 wt%). Indeed, the dilution effect is clear in the CAPEX of the adsorption process, which was nearly three times larger than the thermal reactor cost. Moreover, other devices contribute to the total CAPEX: the PODIC ^®^ reactor and the nanofiltration unit (NF). The power consumption of the PODIC ^®^ reactor is considerable (5.19 kWh/kg _PODIC® _) for this process. Concerning NF, the high salt concentration leads to high osmotic pressures that mean low fluxes through the membrane, which implies large surfaces to treat the involved streams, increasing the total CAPEX.

**Operational cost (OPEX)**

**Figure 2.**
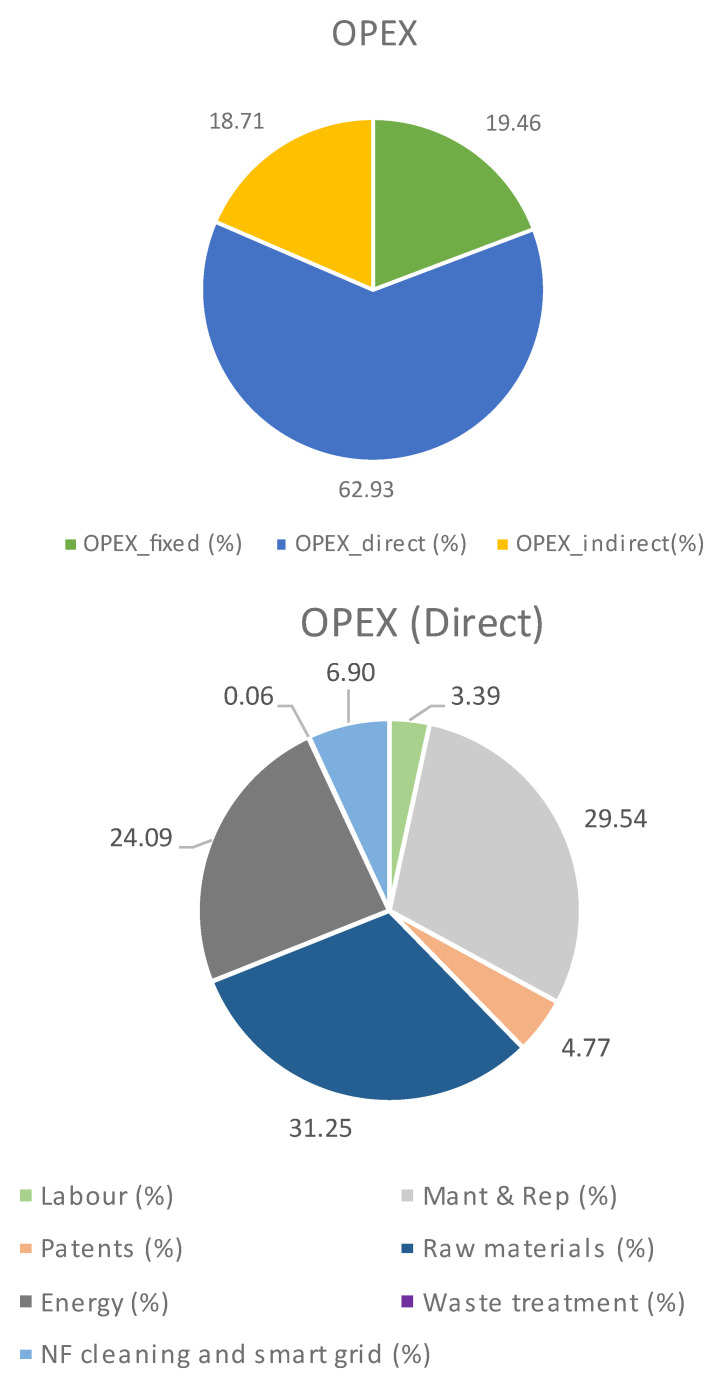
OPEX breakdown when CO _2_ is supplied.

The OPEX for this case can be seen in Table 1 and Figure 2, where it can be observed that the main contributor is the direct cost and, inside it, the raw materials, energy and maintenance have the main roles. As for raw materials breakdown, sodium carbonates account for 69%, while electricity and cooling are the main energy consumed (59% and 33%, respectively). In both cases, PODIC ^®^ production is the main reason for their consumption. Finally, maintenance implies circa 30% of the direct cost, which is explained by the high total CAPEX, which is principally due to the dilution and the investment required for the PODIC ^®^ production.

**Table 1.** Process OPEX when CO _2_ is supplied.

Cost	OPEX, €/kg _Lignin_	OPEX, %
Fixed	0.99	19.46
Direct	3.21	62.93
Indirect	0.96	18.71
Total	5.16	-
Direct OPEX breakdown	OPEX, €/kg _Lignin_	OPEX, %
Labour	0.11	3.39
Energy	0.77	24.09
Raw material	1.00	31.25
Waste treatment	0.00	0.06
Maintenance	0.95	29.54
Patents	0.15	4.77
Membrane cleaning, smart grid, and H _2_ recovery	0.22	6.90

***Analysis of the process without CO _2_ supply***

**Capital cost (CAPEX**)

The evaluation of the investment required for the case when CO _2_ is not fed is presented in ANNEX 1. This approach was included to assess what happens if the permeate obtained from the NF is directly recirculated without an additional reactor to transform the NaOH into carbonate (NaOH reactor). Thus, it was expected to observe a reduction of the CAPEX that could compensate for the expected increase in the OPEX due to the extra carbonate to balance the one that would come from the NaOH. However, the total CAPEX for this approach without CO _2_ supply was similar, even a little higher, to the one with CO _2_ (146 vs 145 M€). This result was because recycling the NaOH implies larger streams at the steady state operation, increasing the size of the equipment, thus the CAPEX. Regarding cost distribution between reaction and separation, both were again balanced. Finally, similarly to the previous case, the high costs are related to dilution, large PODIC ^®^ reactor and NF system.

**Operational cost (OPEX)**

**Figure 3.**
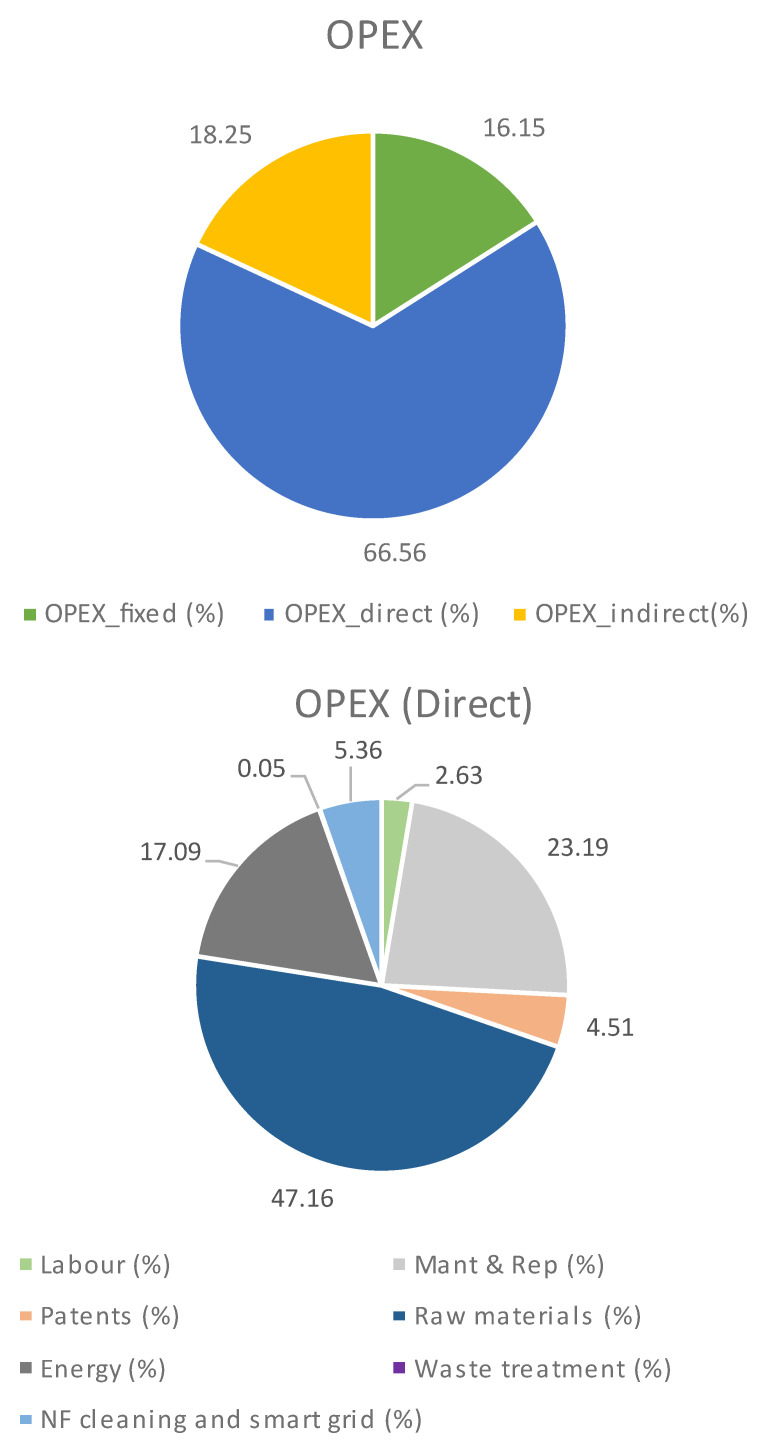
OPEX breakdown when CO _2_ is not supplied.

The values related to the OPEX for this case are listed in Table 2 and Figure 3. As expected, the total OPEX was higher due to the need for more carbonate to produce PODIC ^®^, also explaining the increase in the role of the raw materials in the direct OPEX (carbonate implies 80%). This increase in the raw materials requirements reduced the energy contribution up to 17% being the PODIC ^®^ reactor responsible for circa 64% of the total energy cost. Finally, maintenance contribution is still high due to the dilution.

**Table 2.** Process OPEX when CO _2_ is not supplied.

Cost	OPEX, €/kg _Lignin_	OPEX, %
Fixed	1.00	16.15
Direct	4.13	66.56
Indirect	1.13	18.25
Total	6.27	-
Direct OPEX breakdown	OPEX, €/kg _Lignin_	OPEX, %
Labour	0.11	2.63
Energy	0.71	17.09
Raw material	1.95	47.16
Waste treatment	0.00	0.05
Maintenance	0.96	23.19
Patents	0.19	4.51
NF cleaning and smart grid	0.22	5.36

### Comparison with literature

Once the CAPEX and the OPEX were computed, a comparison with other processes (Cases 1–3) where lignin was transformed into vanillin was performed. Vanillin was selected as a reference since it is the key compound delivered by the process and the one with more information available in the literature. The three cases involve “conventional” approaches, where lignin is thermally degraded by the action of a catalyst, caustic conditions, and oxygen supply. However, while cases 1 and 3 are theoretical studies, case 2 is based on values from a real multiproduct biorefinery when the original raw material is woody biomass. More details can be found in ANNEX 1.

The contrast of all the considered cases in the deliverable can be seen in Table 3, where it can be checked that the best option for the evaluated process is when CO _2_ is supplied. However, the costs for this option are still higher than the ones found in the literature (see ANNEXE 1 for details).

**Table 3.** CAPEX and OPEX in comparison to literature-based cases (see ANNEXE 1 for details).

Value	Process with CO _2_ supply	Process without CO _2_ supply	Case-1	Case-2	Case-3
**CAPEX, €/kg _Vanillin_**	388.57	392.54	120.71	87.7	34.90
**CAPEX, €/kg _Lignin_**	13.76	13.90	0.66	0.58	1.81
**OPEX,€/kg _Vanillin_**	144.22	175.50	24.56	17.67	15.19
**OPEX, €/kg _Lignin_**	5.16	6.27	0.13	0.12	0.79
**(Vanillin) Yield, %**	3.58	3.58	0.55	0.30	5.20

### Improvement options

As previously explained, the highest costs and, thus, profitability bottlenecks, are related to the high dilution in system ^2^ (in addition to PODIC ^®^ reactor and NF costs). For this reason, different process optimisation alternatives (“corrections” to these bottlenecks) were applied in the economic model ^3^ to evaluate how much the cost can be reduced. The corrections were:

a) Assume that the MF/NF is located before the adsorption to reduce the dilution in the separation train. The need for dilution in the thermal reactor to obtain high vanillin yields was experimentally confirmed inside the LIBERATE project. However, it has a detrimental effect on the downstream separation. In principle, water removal by membrane-based ^4^ operations could be performed upstream of the adsorption to avoid losing vanillin, but this could not be experimentally verified. Thus, the effect of this approach (assuming no vanillin losses and a final feasible water content of 35wt%) on the economics was verified by simulation.
b) Free electricity due to national grid green surplus. The process is powered using renewable energies. However, the evaluation involves realistic profiles, meaning that the renewable energy sources' production depends on the weather conditions and, so, they will not be able to produce all the energy required for every hour. Thus, despite having this connection to renewable energy sources, there are hours when energy from the grid is required. For this reason, it was assumed that only during these favourable periods there will be a surplus in the national grid resulting in free renewable energy.
c) Reduce the Thermal reactor residence time to 1 hour to be in line with the ones used as reference cases.
d) Better NF performance. The reported flux for the NF at the working salt concentration was 5 LMH. Nonetheless, this value was measured at a temperature lower than the real one considered in the process (40 °C vs 20 °C). Thus, the real flux could be better and, for this reason, a more optimistic flux of 10 LMH was imposed.

**Figure 4.**
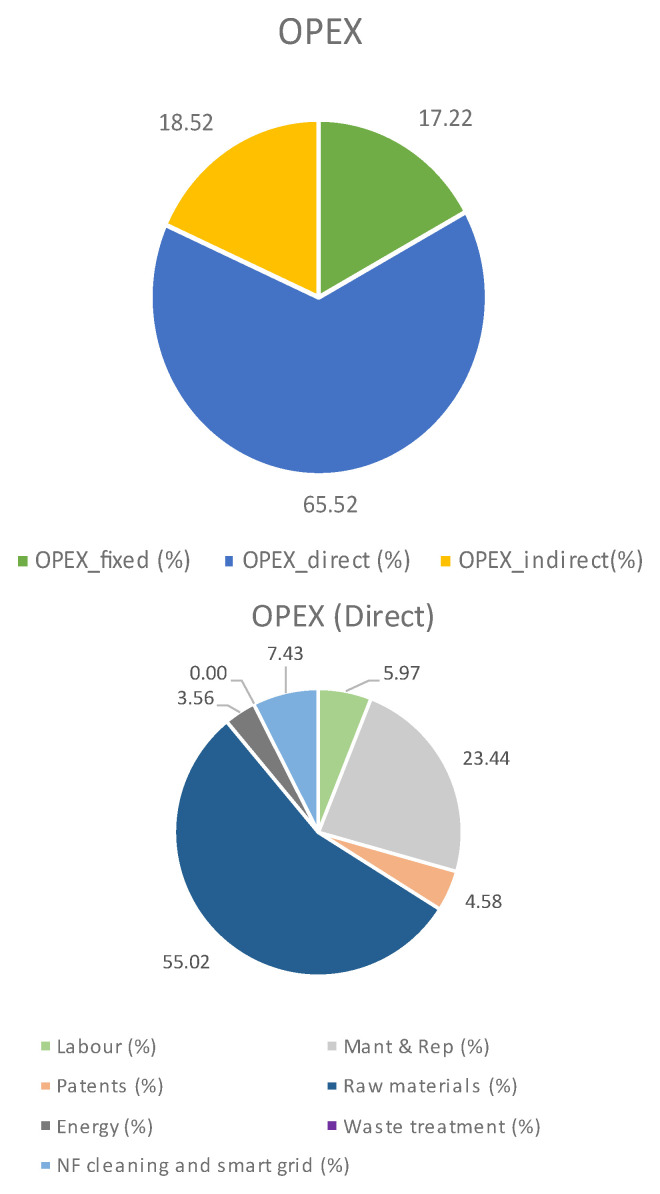
OPEX breakdown when scenarios a, b, c, and d are applied.

The effect of applying all these corrections can be seen in Table 4 and Figure 4, even when all of these improvements are considered, the process costs are closer but still above the ones found in the literature (see ANNEXE 1 for details). Furthermore, the main contribution to the OPEX is, still, the raw materials, where the carbonate required for PODIC ^®^ production implies 69% of the total direct OPEX. Similarly, PODIC ^®^ production represents 28% of the total CAPEX. Therefore, it is obvious that PODIC ^®^ is a bottleneck in reducing the process costs to approach the reference cases. Despite this, it is worth highlighting that reference cases 1 and 2 include assumptions not realistic for the operational conditions used in the evaluated process. And, if these assumptions are included in the economic model ^5^, the vanillin production cost for LIBERATE was: 42.5 €/kg _Vanillin_ (CAPEX) and 23.2 €/kg _Vanillin_ (OPEX), which is similar to those from the reference cases. To sum up, the evaluated process would lead to similar costs to those found in the literature once nearly an ideal performance is assumed (even with the high cost due to the PODIC ^®^ production). However, when a more realistic approach is followed, the costs are in the expected range, but with higher values. **Thus, further efforts would be required on the related chemistry to reduce PODIC ^®^ production costs. Similarly, reducing the dilution in the reaction would be another key factor in enhancing the economic performance**.

**Table 4.** CAPEX and OPEX for different scenarios in comparison to literature-based cases.

Value	Process with CO _2_ supply	a *	a&b *	a&b&c&d *	Case-1	Case-2	Case-3
**CAPEX, €/kg _Vanillin_**	388.57	234.93	234.93	175.10	120.71	87.7	34.90
**CAPEX, €/kg _Lignin_**	13.76	8.32	8.32	6.20	0.66	0.58	1.81
**OPEX,€/kg _Vanillin_**	144.22	116.56	91.87	78.67	24.56	17.67	15.19
**OPEX, €/kg _Lignin_**	5.16	4.13	3.25	2.78	0.13	0.12	0.79
**(Vanillin) Yield, %**	3.58	3.58	3.58	3.58	0.55	0.30	5.20

*Applied scenario(s) based on the corrections explained before.

### Economic indicators

The next step for the analysis consists of computing the economic indicators previously mentioned: Net Present Value (NPV), Internal Rate of Return (IRR), Return On Investment (ROI), and the payback period. This calculation was only done for the best one (the one where CO _2_ is fed) since it is the most probable to imply positive incomes, also including the effect of the different corrections explained in the previous subsection ( Table 5). Considering the results obtained, it can be concluded that the **process is not profitable even under ideal conditions due to the high dilution of the final products, which reduces the selling price**. Further up-grading costs will be imposed to achieve identical product specifications.

**Table 5.** Economic indicators for different scenarios.

Indicator	Process with CO _2_ supply	a&b&c&d *	a&b&c&d * & ideal performance
NPV (€/kg _Lignin_)	-74	-36	-23
IRR (%)	**-**	**-**	**-**
ROI (%)	-	-	-
Payback period (y)	>30	>30	>30

*Applied scenario(s) (see Comparison with literature).

The presented calculations were performed with a Kraft-lignin price of 250 $/t and considering the price index of 2019 to compare with the literature. However, the current Kraft-lignin price should be higher (near 1,000 $/t) ^32, 33^. Further, an estimate using an updated index cost (near 820 for 2022) ^34^ should be evaluated. These re-evaluations and the following calculations will focus on the case without any correction since it represents the “real” process ( Table 6).

**Table 6.** Economic performance with a Kraft-lignin price of 1,000 $/t and an updated index cost.

Value	Process with CO _2_ supply	Process with CO _2_ supply- 1,000	Process with CO _2_ supply -1,000-2022
**CAPEX, €/kg _Vanillin_**	388.57	388.57	497.70
**CAPEX, €/kg _Lignin_**	13.76	13.76	17.62
**OPEX,€/kg _Vanillin_**	144.22	167.10	186.72
**OPEX, €/kg _Lignin_**	5.16	5.91	6.61
**NPV, €//kg _Vanillin_**	-74	-83	-98
**(Vanillin) Yield, %**	3.58	3.58	3.58

### Parametric analysis in terms of the economic indicators

**Figure 5.**
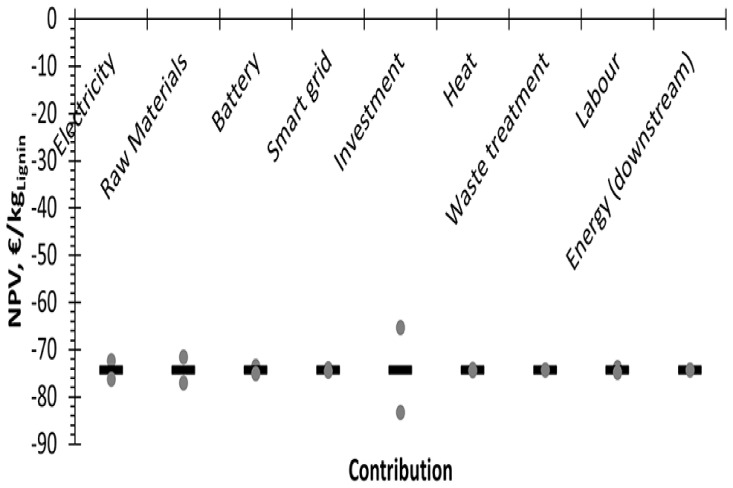
Parametric study of the NPV. Note that the bars refer to the base case, while the dot represents the scenarios with the ±20% changes.

A sensitivity analysis was performed considering a variation of ±20% in the CAPEX and OPEX contributors. However, since no positive incomes were obtained, only the NPV could be assessed ( Figure 5). Focusing on the results, the main factor of cost is the investment cost, whereas raw materials and energy have a less pronounced role. This is in line with the result obtained in the OPEX breakdown for the considered case ( Figure 2), where energy and raw materials had similar contributions to the direct OPEX.

### Process minimum selling prices

Another important indicator of economic performance is the minimum selling price of products (vanillin, phenols, and partially degraded lignin). For a more realistic result, a lignin price of 1,000 $/t was used. An initial screening of five different scenarios was executed to try to explore if positive values are feasible including a change in the scale (the treated lignin flow was increased up to 50 times). Moreover, values for the vanillin selling price close to the maximum feasible value found in the literature (circa 25 $/kg, see ANNEX 1) were used. Similarly, the selling price for the phenols and the partially degraded lignin were set to values in line with the ones found in the literature (see ANNEX 1) ^6^. The results are listed in Table 7, concluding that **only when the scale-up of the process is performed, positive values can be obtained for a realistic evaluation of the process at “feasible” selling prices** (scenario (IV)). Note that all the set prices for this scenario are at high values and, for phenols and partially depolymerized lignin an average of the prices for pure compounds had to be used since lower values would mean a not profitable solution. Similarly, a vanillin selling price much lower than the maximum would also lead to an unprofitable situation. Thus, **the values used in scenario IV are the minimum selling prices**.

**Table 7.** Initial screening of the minimum selling price(s).

Value	Process with CO _2_ supply- 1,000				
	(I)	(II)	(III)	(IV)	(V)
Selling price, $/kg (VAN/PHEN/OLIG) *	7.6/0.76/0.3	25 **/0.76/0.3	25 **/0.76/0.3	25 **/6.7/6.7	7.7/6.7/6.7
Scale (lignin flow), kg/h	1,268.54	1,268.54	50·1,268.54 ***	50·1,268.54 ***	50·1,268.54 ***
NPV, €/kg	-83	-77	-56	2.4	-4
IRR, %	-	-	-	4.13	-
ROI, %	-	-	-	28	-
Payback period, y	-	-	-	29	-

*It refers to the diluted product, not to the pure compound; **Value close to the one used in the references; ***Vanillin output: 18,636 t/y → Nearly 30% global production 2024 ^2^. VAN/PHEN/OLIG: Vanillin/Phenols/Partially depolymerized lignin

### Environmental analysis of the process with and without CO _2_

The analysis of the processes with CO _2_ and without CO _2_ was carried out, evaluating it in the environmental impact categories previously highlighted.

The impacts were determined for the 3 sub-systems referred to the generation of 1 kg of linked product. In the case of the PODIC ^®^ process, it was observed that the case without CO _2_ generated a higher impact than that with CO _2_ in 5 out of 6 impact categories analysed, being equivalent to that obtained with CO _2_ in the impact category of fossil resources scarcity.

Being Lignin Feed subsystem an identical input in both case studies, in the case without CO _2_ the impact of the PODIC ^®^ process generated a higher downstream impact on the production of vanillin and phenolic compounds than in the case study with CO _2_. This is equivalent to an increase in the impact of vanillin production of 2 to 4 % in 4 of 5 environmental impact categories and a decrease of 3 % in case of fossil resource scarcity.

For the case of production of 1 kg of vanillin or phenolic compounds, the lowest environmental impact is for the case with CO _2_, being 1.37 and 1.38 kg CO _2_ eq respectively in the case of global warming, compared to 1.43 and 1.41 kg CO _2_ eq obtained for the case without CO _2_.

**Figure 6.**
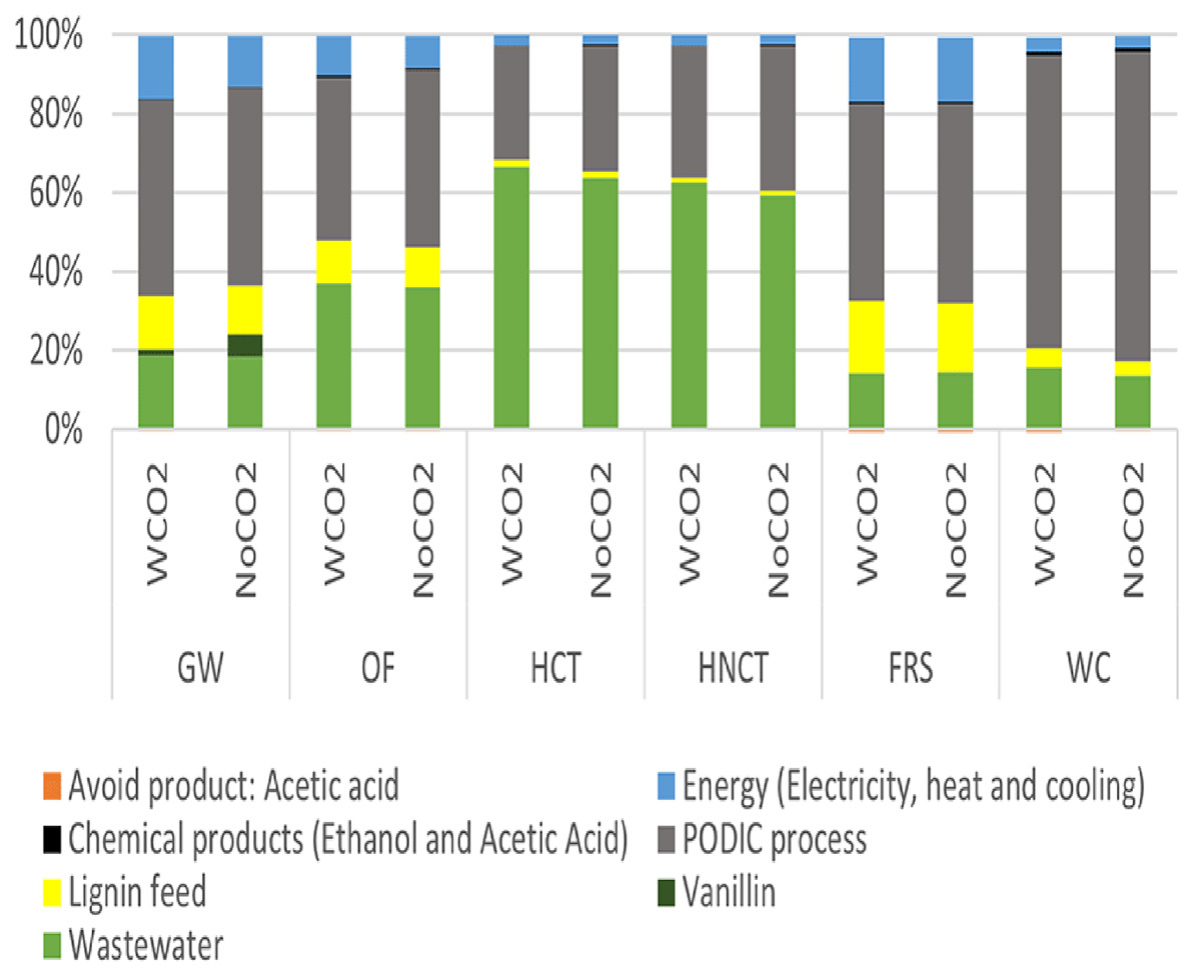
Relative environmental impact analysis of the vanillin production process.

Evaluating both processes to produce 1 kg of vanillin ( Figure 6), it was observed that the PODIC ^®^ process generated the highest environmental impact in 4 of 6 environmental impact categories analysed and the second highest impact in the two Human toxicity environmental impact categories behind wastewater, keeping the same impact ratios for the production of phenolic compounds.

***Causes of the environmental impact***

**Figure 7.**
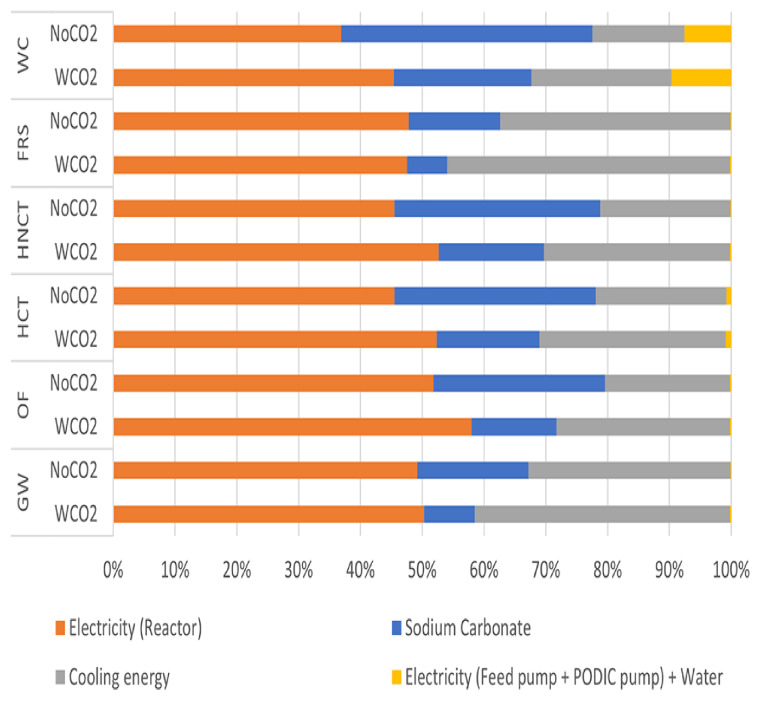
Relative environmental impact analysis of PODIC® production process.

In both cases, it was found that **the electrical consumption of the PODIC ^®^ reactor has the largest impact**. In the case with CO _2_, the largest impact of all categories assessed was due to this electrical consumption. However, in the case without CO _2_, it was observed that the electricity consumption of the reactor had the second highest impact in the environmental impact associated with the water consumption category, after sodium carbonate (see Figure 7).

***Comparison with the literature***

The result obtained for the carbon footprint of producing 1 kg of vanillin was estimated at 1.37–1.43 kg CO _2_ eq. This result is similar to the one reported by LCA on the Borregaard process (1.34 kg CO _2_ eq./kg vanillin) ^35^ and significantly lower than the Kraft-lignin scenario analyzed by Wongtanyawat *et al.*: 137 kg CO _2_ eq./kg vanillin ^23^.

## Conclusions

The proposed process for vanillin production from lignin was successfully modelled and simulated using COCO-simulator, also including the most relevant kinetics and reaction mechanisms (e.g., PODIC ^®^ formation and degradation). Moreover, a complete techno-economic analysis of the proposed process to depolymerize lignin into added-value compounds (e.g., vanillin) was done. During this analysis, the main bottlenecks were identified, namely the high dilution rate and the production of a chemical (PODIC ^®^) to promote the conversion of lignin into vanillin. As an overall conclusion, it can be established that the process can be hardly profitable at the current conditions, even at a larger scale, due to the high costs (CAPEX and OPEX of 13.76 and 5.91 €/kg _Lignin_, respectively).

Finally, based on the results of the environmental analysis, in both cases, the PODIC ^®^ process was the major factor; the highest environmental impact is due to the power consumption of the reactor, giving the option to further reduce this impact by switching to renewable energy-based electricity consumption. The CO _2_ emissions evaluated were similar to those obtained by the *Borregaard* process, again empowering the need for additional improvements in the LIBERATE process to obtain a more sustainable solution.

## Annexes

Annexe 1 can be found at https://doi.org/10.5281/zenodo.8424826, while Annexe 2 is available at https://doi.org/10.5281/zenodo.8424810.

## Nomenclature

*Acronyms*

CAPEX: capital expenditure

IRR: internal rate of return

LCA: life cycle assessment

OPEX: operational expenditure

NPV: net present value

ROI: return on investment

TEA: techno-economic analysis

*Symbols*

A: pre-exponential factor, 1/s

C: concentration of PODIC ^®^, mol/m ^3^

E _a_: activation energy, J

F: Faraday constant, 96,485 s·A/mol

I: the current, A

r _1_: PODIC ^®^ production rate, mol/s

r _2_: PODIC ^®^ decomposition rate, mol/s

V: reaction volume, m ^3^

R: ideal gas constant, 8.31 J/(K·mol)

T: temperature, K

*Greek letters*

η: the current efficiency, dimensionless

ν: the charge count, dimensionless

## Data Availability

Zenodo: Files and inventory, https://doi.org/10.5281/zenodo.8424715 This project contains the following: LCA LCI.xlsx Process model 230212 feedmode2 (1).zip Liberate.pcd TEA Economics_SG.py Economics_Turton.py PV.xlsx P_w.xlsx TEA_2_nco2.py charge.py Data are available under the terms of the Creative Commons Attribution 4.0 International license (CC-BY 4.0).
