# Supplementary material for: Innovative vanillin yielding from lignin: process modelling and assessment
Source: Open Res Eur. 2024 Dec 24;4:5. Originally published 2024 Jan 8. [Version 2] doi: 10.12688/openreseurope.16734.2 (PMC11809479; doi:10.12688/openreseurope.16734.2)
Supplement: Peer review history

# Reviewer report: Reviewer response for version 2

**DOI:** 10.21956/openreseurope.20743.r49127

**Published:** 2024-12-27

**Authors:** Daochen Zhu

**Affiliations:** 1 Jiangsu University, Zhenjiang, China

The quality of the revised manuscript has been greatly improved, and I have no further objections. I suggest accepting it

Is the study design appropriate and does the work have academic merit?

Yes

Is the work clearly and accurately presented and does it cite the current literature?

Yes

If applicable, is the statistical analysis and its interpretation appropriate?

Yes

Are all the source data underlying the results available to ensure full reproducibility?

Yes

Are the conclusions drawn adequately supported by the results?

Yes

Are sufficient details of methods and analysis provided to allow replication by others?

Yes

Reviewer Expertise:

Biodegradation and high-value conversion of lignin

I confirm that I have read this submission and believe that I have an appropriate level of expertise to confirm that it is of an acceptable scientific standard.

# Reviewer report: Reviewer response for version 1

**DOI:** 10.21956/openreseurope.18075.r37851

**Published:** 2024-02-29

**Authors:** Yunxuan Wang

**Affiliations:** 1 The University of Tennesse-Knoxville, Knoxville, Tennesse, USA

The work " Innovative vanillin yielding from lignin: process modeling and assessment " demonstrated the economic and environmental evaluation of vanillin production from lignin using the PODIC ^®^ catalyst. Results show that the proposed process is hard to commercialize, and the use of the proposed PODIC ^®^ led to the most significant environmental impact. The writing of the manuscript hindered the delivery of the study, as many sentences are not grammatically correct and have numerous typos. Overall, the manuscript should be accepted ****after addressing the issues listed below.

1. The manuscript needs spelling checking as there are numerous typos. For example, in the PODIC ^®^ production pathway section the authors wrote: “its production form sodium carbonate had to be modelled”.
2. Full name of an abbreviation should be given for TRL, NPV when they first appear in the context.
3. For Figure 6, the meanings of the bars and dots should be explained.
4. Many citations are missing from the manuscript. The authors made statements like “ones found in the literature” numerous times with no citation and no results from other studies.
5. In the Analysis of the process with CO _2_ supply section, the authors claimed that 145 M is a relatively high cost. What does the “relatively” compare with?
6. In the Introduction, the authors claimed that the use of NaOH is prevented, however, in the study lignin is dissolved in sodium hydroxide, and the process is not NaOH-free.
7. Detailed experimental conditions are missing from the Developed process section.
8. In the Developed process section, the process for “sparging the produced CO _2_ in the caustic solution” to regenerate sodium carbonate needs to be described in more detail.

Is the study design appropriate and does the work have academic merit?

Yes

Is the work clearly and accurately presented and does it cite the current literature?

Partly

If applicable, is the statistical analysis and its interpretation appropriate?

I cannot comment. A qualified statistician is required.

Are all the source data underlying the results available to ensure full reproducibility?

No

Are the conclusions drawn adequately supported by the results?

Yes

Are sufficient details of methods and analysis provided to allow replication by others?

Partly

Reviewer Expertise:

Biorefinery, Lignin valorization, green solvents

I confirm that I have read this submission and believe that I have an appropriate level of expertise to confirm that it is of an acceptable scientific standard, however I have significant reservations, as outlined above.

# Response

**Published:** 2024-12-11

**Authors:** Álvaro Cabeza Sánchez

**Affiliations:** Not Applicable, Spain

We thank the comments and review done to improve the quality of our manuscript. We include the answers below:

- *The manuscript needs spelling checking as there are numerous typos. For example, in the PODIC ^®^ production pathway section the authors wrote: “its production form sodium carbonate had to be modelled”. *
**Answer: ** The complete manuscript was reviewed to remove any typo
- *Full name of an abbreviation should be given for TRL, NPV when they first appear in the context. *
**Answer:** The full name was provided and the text was checked to ensure that the definition was available before being used
- *For Figure 6, the meanings of the bars and dots should be explained. *
**Answer:** Meanings added (the bars refers to the base case and the dot to de case where the 20% change was applied)
- *Many citations are missing from the manuscript. The authors made statements like “ones found in the literature” numerous times with no citation and no results from other studies. *
**Answer:** These references are included in Annexe 1. This was clarified in the text. 
- *In the Analysis of the process with CO _2_ supply section, the authors claimed that 145 M is a relatively high cost. What does the “relatively” compare with? *
**Answer:**  The cost is "high" in comparison to the three reference cases. It was clarified in the text. 
- *In the Introduction, the authors claimed that the use of NaOH is prevented, however, in the study lignin is dissolved in sodium hydroxide, and the process is not NaOH-free. *
**Answer: ** The description was clarified. The evaluated process focuses on the dissolution of the lignin in the PODIC stream, not in externally provided NaOH. In this way, the use of external NaOH is prevented. Indeed, NaOH is a byproduct of the PODIC production. 
- *Detailed experimental conditions are missing from the Developed process section. *
**Answer:** The manuscript is based on the evaluation of the simulated process and a detailed description of the experiments related was not agreed. However, the references to the papers with the experimental work description were included. 
- *In the Developed process section, the process for “sparging the produced CO _2_ in the caustic solution” to regenerate sodium carbonate needs to be described in more detail. *
**Answer:** This step was clarified. Essentially, the idea is to regenerate the Na2CO3 using the produced NaOH and CO2 in the LIBERATE process in a gas-liquid reactor. 

# Reviewer report: Reviewer response for version 1

**DOI:** 10.21956/openreseurope.18075.r37861

**Published:** 2024-02-26

**Authors:** Zhi-Hua Liu

**Affiliations:** 1 Tianjin University, Tianjin, China

Authors provided the modelling of a novel process to promote vanillin production from lignin, including the PODIC© production, lignin depolymerisation and final product separation. The manuscript should be revised to address following concerns.

 1 The references should be updated in the manuscript.

 2. Biosynthesis method of vanillin should also be introduced in the introduction.

 3 More information about vanillin production should be introduced in the introduction section.

 4 The electrochemical PODIC production process should be descripted more clear.

 5 Figure 2 should be revised to clearly show the key information.

 6 Please avoid abbreviations in the text and provide full names

 7 The resolution of some images is too low.

 8 More discussion about the results should be provided.

 9 The key findings of the manuscript should be highlight in each section.

Is the study design appropriate and does the work have academic merit?

Yes

Is the work clearly and accurately presented and does it cite the current literature?

No

If applicable, is the statistical analysis and its interpretation appropriate?

Partly

Are all the source data underlying the results available to ensure full reproducibility?

Yes

Are the conclusions drawn adequately supported by the results?

Yes

Are sufficient details of methods and analysis provided to allow replication by others?

Partly

Reviewer Expertise:

Authors provided the modelling of a novel process to promote vanillin production from lignin, including the PODIC© production, lignin depolymerisation and final product separation. The manuscript should be revised to address following concerns. 1 the references should be updated in the manuscript2. biosynthesis method of vanillin should also be introduced in the introduction3 more information about vanillin production should be introduced in the introduction section4 The electrochemical PODIC production process should be descripted more clear5 Figure 2 should be revised to clearly show the key information6 please avoid abbreviations in the text and provide full names7 The resolution of some images is too low8 more discussion about the results should be provided9 the key findings of the manuscript should be highlight in each section

I confirm that I have read this submission and believe that I have an appropriate level of expertise to confirm that it is of an acceptable scientific standard, however I have significant reservations, as outlined above.

# Response

**Published:** 2024-12-11

**Authors:** Álvaro Cabeza Sánchez

**Affiliations:** Not Applicable, Spain

We would like to acknowledge the review and the comments. Below, we explain how we have addressed them.

- *The references should be updated in the manuscript.* **Answer:** the introduction was reviewed and updated
- *Biosynthesis method of vanillin should also be introduced in the introduction.* **Answer:** references to different approaches (including biosynthesis) were included in the introduction
- *More information about vanillin production should be introduced in the introduction section.* **Answer: ** additional references were included in the introduction
- *The electrochemical PODIC production process should be descripted more clear.* **Answer:**  the description was detailed and references to the related experimental work were included
- *Figure 2 should be revised to clearly show the key information.* **Answer: ** Figure 2 was removed since it is not easily understandable. It can be found in the flowsheet, where it can be used to perform the process simulation
- *Please avoid abbreviations in the text and provide full names.* **Answer: ** The text was reviewed to ensure that the abbreviations are defined before their use and explained to make it easier the reading
- *The resolution of some images is too low.* **Answer:** Images with better quality were provided
- *More discussion about the results should be provided.* **Answer: ** the discussion was reviewed for a better understanding. 
- *The key findings of the manuscript should be highlight in each section.* **Answer:** the key results were highlighted in bold 

# Reviewer report: Reviewer response for version 1

**DOI:** 10.21956/openreseurope.18075.r37853

**Published:** 2024-02-20

**Authors:** Daochen Zhu

**Affiliations:** 1 Jiangsu University, Zhenjiang, China

This manuscript presents an innovative approach to producing vanillin from lignin, utilizing a novel process involving PODIC® for depolymerization. The work includes comprehensive process modeling, techno-economic analysis (TEA), and life cycle assessment (LCA). The present work are interesting, and some comments as follows:

 1. The PODIC® method is interesting, but the introduction to this method is not detailed enough, and please provide a comparative analysis of why you chose this method instead of other depolymerization methods and whether it can provide more advantages. Providing more in-depth analysis and discussion, for example in terms of efficiency, yield, and environmental impact, can help strengthen the persuasiveness of this approach.

 2. The economic evaluation provides a good introduction to the investment and operating costs of the process, and it is best to further discuss how to further reduce costs and improve competitiveness with chemically synthesized vanillin in the future.

 3. This manuscript mainly provides conclusions based on process simulation and evaluation. If the results of small-scale production or experimental data at the laboratory level can be provided, it will provide strong support for the accuracy of model predictions.

Is the study design appropriate and does the work have academic merit?

Yes

Is the work clearly and accurately presented and does it cite the current literature?

Yes

If applicable, is the statistical analysis and its interpretation appropriate?

Yes

Are all the source data underlying the results available to ensure full reproducibility?

Yes

Are the conclusions drawn adequately supported by the results?

Yes

Are sufficient details of methods and analysis provided to allow replication by others?

Yes

Reviewer Expertise:

Biodegradation and high-value conversion of lignin

I confirm that I have read this submission and believe that I have an appropriate level of expertise to confirm that it is of an acceptable scientific standard, however I have significant reservations, as outlined above.

# Response

**Published:** 2024-12-11

**Authors:** Álvaro Cabeza Sánchez

**Affiliations:** Not Applicable, Spain

First of all, we would like to acknowledge the review of our work. The changes and answers of the provided comments are detailed below:

**Reviewer Comment:**

- *The PODIC® method is interesting, but the introduction to this method is not detailed enough, and please provide a comparative analysis of why you chose this method instead of other depolymerization methods and whether it can provide more advantages. Providing more in-depth analysis and discussion, for example in terms of efficiency, yield, and environmental impact, can help strengthen the persuasiveness of this approach. *

**Answer:** The use of PODIC was justified by three main reasons:

1. It means moving from a gas-liquid scenario (thermal oxidation of lignin) to a monophase reactor where electrochemically produced PODIC® is used. Thus, mass transfer limitations would be avoided.
2. The base fact that PODIC® selectively oxidizes lignin into vanillin
3. It implies an electrochemical approach where only electrons are used as reagents and thus toxic and/or harmful reagents can be omitted/reduced

The manuscript focused on the evaluation of the economic and environmental performance of the optimal solution found in the LIBERATE-process at pilot scale (TRL6). For this reason, the obtained results were just compared with the literature in terms of cost and environmental impact. Other evaluations would require the use of experimental information that was not agreed to share in this document. However, the papers where the experimental data are available and discussed were included in the manuscript. 

 -------------------------------------------

**Reviewer Comment:**

- *The economic evaluation provides a good introduction to the investment and operating costs of the process, and it is best to further discuss how to further reduce costs and improve competitiveness with chemically synthesized vanillin in the future. *

**Answer:** The process bottlenecks were identified (mainly dilution and the PODIC production) and feasible solutions were provided and evaluated. Moreover,  process optimisation was also done during the scale-up. All of them have been grouped inside a subsection named "Improvement options" for a better understanding of the discussion. 

 -------------------------------------------

**Reviewer Comment:**

- *This manuscript mainly provides conclusions based on process simulation and evaluation. If the results of small-scale production or experimental data at the laboratory level can be provided, it will provide strong support for the accuracy of model predictions. *

**Answer:** ****The manuscript focuses on giving a view of the feasible performance at an industrial scale based on simulation. In this way, other researchers can get access to the overall aspects of the solution developed and the “current” economic and environmental perspectives. The experimental part is not in the scope and, indeed, it was not agreed to include it here since it was published by the developers in other papers. The references of these experimental papers were included.

# Reviewer report: Reviewer response for version 1

**DOI:** 10.21956/openreseurope.18075.r37489

**Published:** 2024-02-13

**Authors:** Adam Slabon

**Affiliations:** 1 University of Wuppertal, North Rhine-Westphalia, Germany

The manuscript of Sanchez et al reports the chemical process to obtain vanillin from lignin based on process modelling. The work is interesting for both academic and industrial researchers, but there are three issues.

 1. there is no experimental proof that vanillin can be obtained in such high yield as required for the process after scale-up. The authors should take the low yield into account or provide experimental proof for the reactor concept. I do not ask for building the reactor, but the difficulties for scale-up regarding yield should be discussed.

 2. the references omit the major developments in the field of electrochemical lignin depolymerization. For instance, using carbonate electrolytes have been recently reported to be efficient solvent systems ref[1]. There are several more recent advancements in the field. The introduction is short and could be extended. 

 3. What about the stability of the electrodes during long-term electrolysis? Nearly all published articles just focus on relatively short time frames. However, for many catalyst systems the leaching of the electrode provides many problems.

Is the study design appropriate and does the work have academic merit?

Yes

Is the work clearly and accurately presented and does it cite the current literature?

No

If applicable, is the statistical analysis and its interpretation appropriate?

Yes

Are all the source data underlying the results available to ensure full reproducibility?

Partly

Are the conclusions drawn adequately supported by the results?

Partly

Are sufficient details of methods and analysis provided to allow replication by others?

No

Reviewer Expertise:

electrocatalysis

I confirm that I have read this submission and believe that I have an appropriate level of expertise to confirm that it is of an acceptable scientific standard, however I have significant reservations, as outlined above.

## References

[1] : Organic Chemicals from Wood: Selective Depolymerization and Dearomatization of Lignin via Aqueous Electrocatalysis. *ChemSusChem* .2024; 10.1002/cssc.202301617 e202301617 10.1002/cssc.202301617 38179850

# Response

**Published:** 2024-12-11

**Authors:** Álvaro Cabeza Sánchez

**Affiliations:** Not Applicable, Spain

First of all, we would like to thank the review and the provided comments. Below, we explain how we have addressed them:

- *There is no experimental proof that vanillin can be obtained in such high yield as required for the process after scale-up. The authors should take the low yield into account or provide experimental proof for the reactor concept. I do not ask for building the reactor, but the difficulties for scale-up regarding yield should be discussed. * 

**Answer:** The aim of the research paper is not focused on the experimental work but on the evaluation of the technology developed inside the LIBERATE-project at a relevant scale. Thus, the idea is to provide insights into the expected economic and environmental performance of the LIBERATE approach and investigate whether it could be translated into an industrial environment by evaluating the main burdens. In this way, other researchers can get an idea of the “current” performance of this lignin valorisation approach. On the other hand, it is true that the scale implemented in the model cannot be substantiated with experimental data from a plant of the same capacity. However, in the course of the LIBERATE-project, a scale-up from lab to pilot scale (TRL6) was done by SINTEF obtaining similar yields in both scales. Thus, it was demonstrated that the technology could be used at different scales without significant losses in the yields. Furthermore, in the evaluation, scale-up approaches (like numbering for the electrochemical reactor) were included to have a more realistic evaluation. The reference to the related paper was included in the document.

- The references omit the major developments in the field of electrochemical lignin depolymerization. For instance, using carbonate electrolytes have been recently reported to be efficient solvent systems ref[1]. There are several more recent advancements in the field. The introduction is short and could be extended. 

**Answer:** As previously mentioned, the work focused on the evaluation of the technology and not on the experimental part. Thus, the literature review was focused on those works where economic/environmental data were provided. However, we references on the field of electrochemical lignin depolymerization accordingly.

- *What about the stability of the electrodes during long-term electrolysis? Nearly all published articles just focus on relatively short time frames. However, for many catalyst systems the leaching of the electrode provides many problems.* 

**Answer:** The electrochemical cell for PODIC production was opened at the end of the project and the state of the electrodes was evaluated. However, the authors of this paper are not the owners of this information and the results were not made public by the manufacturer. Thus, if someone is interested in further work on these electrodes, we recommend directly contacting the electrochemical cell manufacturer (CONDIAS, https://www.condias.de/en-gb)
